# Overview of Oncology: Drug-Induced Cardiac Toxicity

**DOI:** 10.3390/medicina61040709

**Published:** 2025-04-12

**Authors:** Nilima Rajpal Kundnani, Vincenzo Passini, Iulia Stefania Carlogea, Patrick Dumitrescu, Vlad Meche, Roxana Buzas, Daniel Marius Duda-Seiman

**Affiliations:** 1 University Clinic of Internal Medicine and Ambulatory Care, Prevention and Cardiovascular Recovery, Department VI—Cardiology, “Victor Babes” University of Medicine and Pharmacy, 3000041 Timisoara, Romania; knilima@umft.ro (N.R.K.);; 2 Research Centre of Timisoara Institute of Cardiovascular Diseases, “Victor Babes” University of Medicine and Pharmacy, 3000041 Timisoara, Romania; 3 Faculty of Medicine, “Victor Babes” University of Medicine and Pharmacy, 3000041 Timisoara, Romania; 4 1st Medical Semiology, Internal Medicine, Department V, “Victor Babes” University of Medicine and Pharmacy, 3000041 Timisoara, Romania; 5 Center for Advanced Research in Cardiovascular Pathology and in Hemostaseology, “Victor Babes” University of Medicine and Pharmacy, 3000041 Timisoara, Romania

**Keywords:** cardiotoxicity, chemotherapy, radiotherapy, anthracyclines, anti-HER2 receptor monoclonal antibody

## Abstract

Cancer medications can cause cardiac issues, which are difficult to treat in oncologic patients because of the risk of complications. In some cases, this may significantly impact their well-being and treatment outcomes. Overall, these complications fall under the term “drug induced cardiotoxicity”, mainly due to chemotherapy drugs being specifically toxic to the heart, causing a decrease in the heart’s capacity to pump blood efficiently and leading to a reduction in the left ventricular ejection fraction (LVEF), and subsequently possibly leading to heart failure. Anthracyclines, alkylating agents, and targeted therapies for cancer hold the potential of causing harmful effects on the heart. The incidence of heart-related issues varies from patient to patient and depends on multiple factors, including the type of medication, dosage, duration of the treatment, and pre-existing heart conditions. The underlying mechanism leading to oncologic-drug-induced cardiovascular harmful effects is quite complex. One particular group of drugs, called anthracyclines, have garnered attention due to their impact on oxidative stress and their ability to cause direct harm to heart muscle cells. Reactive oxygen species (ROS) cause harm by inducing damage and programmed cell death in heart cells. Conventional biomarkers alone can only indicate some degree of damage that has already occurred and, therefore, early detection is key. Novel methods like genetic profiling are being developed to detect individuals at risk, prior to the onset of clinical symptoms. Key management strategies—including early detection, personalized medicine approaches, and the use of novel biomarkers—play a crucial role in mitigating cardiotoxicity and improving patient outcomes. Identification of generated genetic alterations and the association to an increased likelihood of cardiotoxicity will allow treatment in a more personalized approach, aiming at decreasing rates of cardiac events while maintaining high oncological efficacy. Oncology drug-induced cardiotoxicity is managed through a combination of preventive strategies and therapeutic interventions from the union of cardiac and oncological knowledge.

## 1. Introduction

### Importance of Recognizing Cardiotoxicity in Cancer Treatment

Taking into consideration the cardiotoxic effects of the drugs used in cancer treatment marks the beginning of enhancing quality of life. Chemotherapies, radiotherapies, and other anticancer modalities can cause significant cardiovascular complications. Treatment-related cardiotoxicity is associated with a high morbidity and mortality burden observed in cancer survivors [1,2]. Recent studies indicate a significant rise in cardiotoxic events among cancer survivors, emphasizing the urgent need for improved cardiac monitoring.

The prevalence of cardiotoxicity varies depending on the type of cancer treatment used. For instance, in patients treated with anthracyclines, cardiomyopathy is found in approximately 1–26%, but this effect may be compounded by the concurrent use of trastuzumab (a humanized anti-HER2 receptor monoclonal antibody) [2]. One recent study indicated that as many as 25% of all cancer patients might be affected by some form of cardiotoxicity, and survivors of pediatric cancers experienced a cardiac mortality risk that is almost eight times larger compared to the general population [1,2]. This underscores the evolution of cancer treatments and the importance of recognizing cardiotoxicity early in the treatment continuum. A proper definition of chemotherapy-induced cardiotoxicity is yet to be established [3].

To manage various cardio-toxic effects of the drugs, a multidisciplinary approach is required between oncologists and cardiologists. The current guidelines suggest “dynamic partnerships”, where both specialties are required to work closely together to monitor patients at risk and provide preventive strategies [4]. Despite the recognition of this need, many patients are not referred to cardiology services until after they develop symptoms of cardiotoxicity. Studies have reported that only 15% of patients were referred to a cardiologist before the administration of chemotherapy, revealing a serious, significant gap in the standard of preventive practice [1,4]. With the availability of novel cancer therapies, there arises a constant need for continuous monitoring of the patients in order to detect possible adverse effects on the vital organs.

## 2. Background on Cancer Treatments: Evolution of Chemotherapy and Targeted Therapies

Advancements in chemotherapy and targeted treatments have significantly transformed the landscape of cancer treatment in the last five decades by converting aggressive cancers from being rapidly fatal to being more effectively manageable by oncologists’ long-term care strategies. The emergence of chemotherapy as a treatment option in the twentieth century marked a revolutionary breakthrough in cancer therapy by employing cytotoxic agents that target and eliminate both fast-growing normal cells and cancerous cells. The approach often led to side-effects and proved ineffective in the long run, as the cancer cells developed resistance to the treatments [5,6].

In the 1980s, there was a shift towards using a combination of chemotherapy treatments for enhancing effectiveness to combat resistance in cancer therapy. The transition towards targeted classes such as interferons and retinoids marked a significant advancement in treatment strategies [7].

Since then, the field of oncology has seen great progress thanks to advancements in biology and genetics, which allowed for the identification of specific genetic changes that differentiate normal cells from cancerous ones, and the characterization of key molecular alterations that trigger cancer growth. An example of this progress is the development of imatinib in 2001 as targeted therapy for patients with chronic myeloid leukemia (CML), marking a major breakthrough in targeted treatment by focusing on specific genetic mutations instead of using harmful genotoxic substances to improve patient outcomes. These modifications also notably reduced the levels of side effects while simultaneously improving the survival rates [5,6].

It is important to note that while these treatments have improved survival, they have also introduced cardiotoxic risks. For example, immune checkpoint inhibitors have been linked with immune-mediated myocarditis and other cardiac complications. A systematic review and meta-analysis conducted by Nielson DL et al. highlighted the importance of early detection of cardiovascular adverse effects of immune checkpoint inhibitors while stating that in some cases, ceasing their use and initiating corticosteroids can prove beneficial [8].

Moreover, along with the development in cancer treatment through immunotherapy, the usage of immune checkpoint blockers and monoclonal antibodies has recently been on the rise as well. These treatments leverage the body’s natural defense mechanisms to combat cancer efficiently by pinpointing and eliminating cancerous cells. Tailored therapies have shown promising outcomes in terms of both survival rates, and in enhancing the quality of life for individuals diagnosed a decade ago with breast cancer that tested positive for human epidermal growth factor receptor 2 (HER2 positive) [9,10]. Although overall survival rates have improved, these benefits are tempered by an increased incidence of cardiac complications, making cardiac surveillance essential [11]. The extended life span achieved with the help of modern oncology treatment drugs on hand helps deal with cancers more efficiently, but on the other hand, it increases the burden of cardiovascular pathologies, which are either due to the long-term usage of drugs or can be age-related [12]. They predispose patients to late-onset cardiovascular complications such as heart failure, arrhythmias, and coronary artery disease. Hence, there exists an increasing clinical need for cardio-oncology programs to address these risks proactively.

Worldwide medical research is being conducted to obtain a better understanding of gene mapping technologies. Drug resistance remains a challenge despite advancements in the field. Efforts are underway to explore alternative therapies, like chimeric antigen receptor (CAR) T cell therapy, known for its efficacy in treating hematological cancers [13].

### 2.1. Patient Survival Rates and Quality of Life

Throughout the years, advancements in cancer treatment have increased the chances of survival for cancer patients. However, the impact of these treatments on the quality of life for patients is still uncertain. Due to advancements in cancer detection and therapies, there is a probability of individuals surviving cancer [14]. For example, the overall five-year survival rate for all types of cancers has increased from 50% in the mid-seventies to 67%. Both negative impacts and receiving adequate support can affect a person’s quality of life, not only during their journey as a cancer survivor but also throughout the entire spectrum of cancer care [15,16].

Over the past few decades, cancer cures have enhanced the overall survival rate, but their effects on the quality of life are complex. Cancer diagnosis, therapies, and treatment have received increased attention over the years, providing patients with a higher probability of surviving cancer [17].

Numerous individuals who have overcome cancer often mention experiencing symptoms that affect their well-being and various aspects of their lives. When examining fatigue in cancer survivors according to four study outcomes, its occurrence was observed with rates ranging from 50 to 90% in early stages, 25% in those in intermediate stages, and between 40 and 50% for advanced cases. Fatigue is noted to persist during cancer therapy and afterwards in about 30 to 40% of survivors [18]. Patients may also face challenges such as pain issues, challenges with cognitive function post chemotherapy (referred to as “chemo brain”), sexual difficulties, and disruptions in sleep patterns [18]. These symptoms can significantly impact a person’s job performance and relationships.

The type of treatment given and the dosage can have an impact on the well-being of individuals with cancer. For example, those undergoing chemotherapy often experience more side effects and disruptions in their quality of life compared to those receiving targeted therapies or immunotherapy [19]. Similarly, surgeries that aim to treat cancer can sometimes lead to effects in terms of both function and physical appearance in the long run, especially when they involve visible body parts or vital organs, like the breast, head, or neck [19].

Survivorship care for cancer patients has developed as a part of cancer treatment strategy with an emphasis on managing the long-term effects and ensuring a high quality of life for survivors. This includes addressing well-being taking into consideration as well as its mental and social aspects [20]. Programs promoting wellness through activities like exercise programs and cognitive therapy along with medical services are shown to improve aspects of the quality of life for cancer survivors [20].

The majority of cancer survivors claim improvements in the outlook of life, relationships and personal development after cancer experience, which is called post-traumatic growth [18]. This demonstrates the recovery ability of many cancer survivors and the capability of achieving positive psychological changes regardless of unfavorable situations.

### 2.2. Definition of Cardiotoxicity: Variability in Definitions and Clinical Implications

Cardiotoxicity describes the side effects of cancer treatments on the heart and circulatory system. These effects range from unapparent changes in cardiac function to potentially fatal heart failure. The primary objective clinical definition of cardiotoxicity uses changes in the left ventricular ejection fraction (LVEF). There have been many proposed thresholds defining cardiotoxicity using changes in LVEF [21].

The Cardiac Review and Evaluation Committee (CREC) defines cardiotoxicity as follows:

1. Concomitant signs or symptoms of congestive heart failure CHF characterized by a reduction in LVEF of at least 5% to below 55%.
2. LVEF of at least 10% decrease to below 55% in the absence of clinical signs or symptoms [22].

Other definitions have been proposed, including the following:

- A drop of LVEF by a value of >10 percentage points to a value of <50%.
- >20 percentage points absolute decrease in LVEF.
- Any LVEF declines to <50%.

Cardiotoxicity onset can also occur at significantly different times.

Early onset: This may present acutely during treatment or within the first year after treatment was discontinued (early based on blood count findings alone) [23].

Late onset: This occurs more than a year after treatment was completed (secondary to bone marrow aplasia) [1]. The added variability in timing further complicates long-term monitoring and follow-up strategies for cancer survivors.

While LVEF-based definitions are widely used, they have several limitations. Based on the severity of the LVEF, different clinical signs and symptoms can develop leading to poor QOL. For instance, reading of LVEF changes may not detect early, subclinical myocardial dysfunction that later develops into symptomatic heart failure [22]. Loading conditions can affect LVEF measurements and may not be representative of intrinsic myocardial contractility. Without meeting the LVEF-based criteria for cardiotoxicity [21], some patients may develop significant cardiovascular complications.

Because of these limitations, there has been a growing interest in other markers of cardiac dysfunction including global longitudinal strain (GLS) and cardiac biomarkers like troponin and natriuretic peptides.

### 2.3. Cardiotoxicity Mechanism and Types

#### 2.3.1. Mechanisms of Cardiotoxicity

Cardiotoxicity due to oncologic drugs is diverse and often multifactorial.

Anthracyclines: For anthracyclines, one of the most well-studied classes of cardiotoxic chemotherapeutics, several mechanisms have been identified: oxidative stress, mitochondrial dysfunction, DNA damage, calcium dysregulation, apoptosis induction, and disruption of normal cellular signaling pathways (Table 1).

- Oxidative Stress: Cardiac myocytes are similarly damaged by oxidative damage from the generation of reactive oxygen species and free radicals by anthracyclines [24].
- Mitochondrial Dysfunction: These drugs could influence their mitochondrial function and, thus, energy production in the cardiomyocytes [24].
- DNA Damage: Anthracyclines may intercalate into DNA synthesis, causing breaks and inhibition of DNA repair mechanisms [24].

Additionally, genome editing, which enables precise and highly reproducible genome manipulation, has enabled the study of disease genetics and pathogenesis, and the development of targeted human therapeutics.

- Calcium Dysregulation: Chemotherapy with anthracyclines can affect calcium homeostasis, causing cardiac contractility [24].
- Apoptosis Induction: Programmed cell death can be triggered in cardiomyocytes by anthracyclines [24].

Other drugs have different molecular mechanisms of cardiotoxicity, as follows:

- Tyrosine kinase inhibitors: tyrosine kinase inhibitors can disrupt important normal cellular signaling pathways needed for cardiac function [25]. Imatinib, for example, inhibits the BCR-ABL tyrosine kinase and other kinases, including c-Abl, which play crucial roles in cardiac function. Inhibition of c-Abl can disrupt mitochondrial integrity and function, leading to cardiomyocyte apoptosis and subsequent cardiac dysfunction [26].

Additionally, imatinib has been shown to induce endoplasmic reticulum (ER) stress and inflammation in cardiomyocytes, further contributing to its cardiotoxic effects [27].

Moreover, imatinib can interfere with autophagic processes by accumulating in lysosomes and blocking the fusion of autophagosomes and lysosomes, resulting in impaired cellular homeostasis and cardiotoxicity [28].

- Immune checkpoint inhibitors: Another notable class of antitumoral drugs, immune checkpoint inhibitors (ICIs), specifically inhibitors of anticytotoxic T-lymphocyte associated antigen 4 and anti-PD/PD-L1 (anti-programmed death receptor and receptor ligand-1), are the mainstay of treatment for many malignancies. The mechanism of immune-related adverse events is mainly related to the aberrant activity of autoreactive T cells, which leads to autoimmune inflammatory reactions affecting both on-target and off-target organs [29]. Immune-related adverse events have variable presentations, ranging from asymptomatic laboratory findings to fulminant life-threatening diseases. There are specific guidelines for treating these immune-related adverse events, depending on the severity of toxicity (graded from 1 to 4) [30].

These ICIs refer to specialized monoclonal antibodies that have the ability to boost the body’s immune response against cancer by interfering with immune system regulators known as down-regulators. These down-regulators include PD-1 and CTLA-4, as well as their associated ligand, PD-L1. By blocking these immune checkpoints, ICIs effectively unleash the activity of effector T-cells, thus facilitating a more robust anti-tumor response. When T-cells are activated, the CTLA-4 activation occurs. Activated T-cells and a specific subgroup of CD25+ CD4+ T-cells known as T-regulatory (T-reg) cells both exhibit the presence of CTLA-4. Being a member of the immunoglobulin supergene family, CTLA-4 shares about 30% similarity with CD28. The affinity and avidity of its binding to CD80/86 are significantly greater than that of CD28. The binding of CTLA-4 to CD80/86 leads to the suppression of T-cell-mediated immune responses. This occurs through the reduction in IL-2 and IL-2 receptor expression, ultimately resulting in a decrease in overall immune activity. Additionally, CTLA-4 can also influence immunity through its impact on T-reg cells [31].

There are distinct differences between the regulation of T cells through the PD-1-PD-L1 axis and that of CTLA-4. PD-1, a component of the immunoglobulin superfamily, becomes activated in peripheral T cells and B cells upon stimulation. Its primary role is to maintain peripheral tolerance. PD-1 engages with two ligands, namely PD-L1 and PD-L2, within the peripheral tissues. PD-L1 can be found in B cells, macrophages, T cells, and dendritic cells when they are in a resting state. The expression of PD-L2 is uncommon in quiescent immune cells; however, pro-inflammatory cytokines can stimulate its synthesis. The activation of both PD-1 and CTLA-4 pathways ultimately impacts the Akt signaling pathway, however, the specific pathways and outcomes of antibody inhibition vary. Akt, also known as protein kinase B (PKB), plays a pivotal role in regulating important cellular functions, including metabolism, programmed cell death (apoptosis), and cell proliferation. The CD28 binding in T cells induces the activation of phosphatidylinositol 3-kinase (PI3K), which then associates with Akt, leading to its phosphorylation. While PD-1 signaling directly counteracts PI3K, CTLA-4 exerts its effects through the activation of PP2A, a phosphatase. Overall, these findings serve to emphasize the distinctions in the effects of anti-PD-1/PD-L1 and anti-CTLA-4 antibodies on T cells in relation to their activation stage, downstream pathways engaged, and site of action.

Currently, the FDA has granted approval to numerous ICIs for the management of diverse forms of cancer. These include ipilimumab (anti-CTLA-4), nivolumab, pembrolizumab, and cemiplimab (anti-PD-1), as well as avelumab, atezolizumab, and durvalumab (anti-PD-L1). With the ability to hinder the interactions between PD-L1 and PD-L2, anti-PD-1 agents hold promise. However, it has been noted that specific anti-PD-1 and anti-PD-L1 agents exhibit variations in terms of autoimmune toxicity. Numerous studies have unequivocally shown that the efficacy of a PD-1 blockade and a PD-L1 blockade in diminishing tumor growth is essentially identical.

The most alarming side effect of ICIs is the occurrence of myocarditis [8]. Vascular dysfunction, hypertension, and thromboembolism can also occur.

- Anti-angiogenic drugs: another class of anti-tumoral drugs, called anti-angiogenic drugs, particularly those targeting the vascular endothelial growth factor (VEGF) pathway, have been instrumental in cancer therapy but are associated with cardiotoxic effects through various mechanisms. Inhibition of VEGF signaling can lead to hypertension, as VEGF plays a crucial role in maintaining endothelial function and nitric oxide production; its inhibition results in vasoconstriction and elevated blood pressure [32].

Additionally, VEGF inhibitors can cause left ventricular dysfunction and heart failure by inducing microvascular rarefaction, reducing myocardial capillary density, and impairing cardiac perfusion. Furthermore, these agents may promote thromboembolic events due to endothelial dysfunction and a pro-coagulant state [33].

In contrast, drugs from the same class targeting the placental growth factor (PlGF) pathway may exhibit a different cardiotoxicity profile. PlGF is involved in pathological angiogenesis, and its inhibition has been explored as a therapeutic strategy with potentially fewer cardiovascular side effects. Studies suggest that anti-PlGF therapies might avoid some of the hypertension and thrombotic complications associated with VEGF inhibition, as PlGF is less involved in maintaining normal vascular homeostasis. However, comprehensive clinical data are limited, and further research is necessary to fully elucidate the cardiovascular safety profile of anti-PlGF therapies [34].

#### 2.3.2. Types of Cardiotoxicity

Cardiotoxicity is a monumental problem in the treatment of cancer because many cancer drugs can have adverse effects on the heart. Cardiotoxicity can vary considerably depending on the type of drug and the patient factors involved as well as on the mechanisms, timing, and types of disease [35] (Table 2).

- Left Ventricular Dysfunction and Heart Failure: This is the most prevalent and known manifestation, shared with anthracyclines (and some targeted therapies) [24,25].
- Arrhythmias: General chemotherapeutics can affect the complex rhythms, from benign to life-threatening [25].
- Myocardial Ischemia: For example, fluoropyrimidines can induce coronary vasospasm, resulting in ischemia [25].
- Hypertension: VEGF inhibitors have specifically been shown to exacerbate (or in some cases, cause) hypertension [25].
- Thromboembolism: Some chemotherapies are associated with an increased risk of thromboembolic events [25].

Pericardial Disease: Pericarditis or pericardial effusion [25] also from certain drugs.

### 2.4. Acute vs. Chronic Cardiotoxicity

The timing of cardiotoxicity onset is an important consideration in cancer treatment.

#### 2.4.1. Acute Cardiotoxicity

Acute cardiotoxicity occurs during or shortly after treatment (within 2 weeks) [24].

It usually manifests as transient arrhythmias, pericarditis, or acute left ventricular dysfunction [24]. Most are usually reversible and not dose dependent [24]. It is also notable that anthracyclines can be seen with immediate effects like arrhythmias or myocarditis [36].

#### 2.4.2. Chronic Cardiotoxicity

Chronic cardiotoxicity may be further divided into early onset (<1 year) and late onset (>1 year after treatment). The most common form is early onset chronic cardiotoxicity, typically dilated cardiomyopathy [24]. Late-onset can occur years or even decades after the treatment is complete [24]. The irreversible complications often concur with a poorer prognosis [24], especially for anthracyclines [24], where the effect is dose-dependent.

Recent findings are challenging traditional classification of the acute, early on chronic, and late on chronic cardiotoxicity. According to these researchers, anthracycline-induced cardiotoxicity may now be viewed as a continuum from subclinical myocardial cell injury to asymptomatic left ventricular dysfunction to symptomatic heart failure if unidentified untreated [24].

### 2.5. Immediate Effects Versus Long-Term Consequences

Cardiotoxicity induced by oncology drugs is complex and polyphasic, combining immediate effects and long-term consequences.

### 2.6. Cardiotoxicity Pathways

Oncology drugs can cause cardiotoxicity through various mechanisms:

- Oxidative Stress: Reactive oxygen species (ROS) produced by many chemotherapeutic agents (e.g., anthracyclines) have been shown to damage cellular components (proteins, lipids, DNA). Cardiomyocyte death can result from this oxidative stress and a malfunctioning mitochondrial system [24].
- Mitochondrial Dysfunction: Anthracyclines are used as drugs that directly impair mitochondrial function to reduce energy production in cardiomyocytes closing cellular dysfunction or death [24].
- DNA Damage: Anthracyclines are drugs which intercalate with DNA and create breaks in the DNA, disturbing DNA repair mechanisms. Apoptosis or senescence of cardiac cells [27].
- Calcium Homeostasis Disruption: Chemotherapeutics can alter calcium handling in cardiomyocytes, thus reducing contractility and potentially causing arrhythmias [22].
- Vascular Toxicity: Consequently, certain targeted therapies such as VEGF inhibitors may lead to endothelial dysfunction and indeed hypertension, in turn affecting cardiac function [37].
- Immune-Mediated Damage: Immune mediated myocarditis downstream of immune checkpoint inhibitors may result in severe cardiac complications [37].

All of these will be elaborated in their own specific paragraph.

#### 2.6.1. Short-Term vs. Long-Term Consequences

The cardiotoxic effects of oncology drugs can be categorized into immediate effects and long-term consequences.

#### 2.6.2. Short Term Effects

1. Acute Arrhythmias: Some drugs, such as anthracyclines, can provoke immediate rhythm disturbances, from benign to lethal [22].
2. Acute Left Ventricular Dysfunction: The activity can occur shortly after or during treatment, and tends to reverse when the drug is discontinued [24].
3. Myocarditis: Acute myocarditis has been particularly associated with immune checkpoint inhibitors and appears as early as weeks after treatment initiation [37].
4. Acute Hypertension: Hypertension related to VEGF inhibitors can occur rapidly and may need to be managed immediately [37].

#### 2.6.3. Long-Term Consequences

Chronic Cardiomyopathy: Left ventricular function can progress to heart failure, many years after the end of treatment in the setting of progressive degeneration (anthracyclines or some targeted therapies) [22,24].

Accelerated Coronary Artery Disease: In some cases, the therapies may in fact accelerate atherosclerosis and increase the long-term risk for coronary events [22].

Valvular Heart Disease: Radiation therapy in combination with chemotherapy tend to cause progressive valvular dysfunction over time [22].

Subclinical Cardiac Dysfunction: Long-term survivors may have subclinical cardiac abnormalities leading to future cardiovascular events and with or without overt clinical symptoms [24].

Secondary Cardiovascular Risk Factors: Long-term cardiovascular risk to Cancer survivors may be further increased by metabolic abnormalities (e.g., hypertension, dyslipidemia) [19].

### 2.7. Reversible vs. Irreversible Cardiotoxicity

Historically, cardiotoxicity has often been categorized into two main types:

1. Type I (Irreversible): This type had typically been linked with anthracyclines, and was thought to have caused permanent cardiac damage. Traditionally, cardiac dysfunction previously believed to be basically irreversible and having a poor prognosis [38].
2. Type II (Reversible): This type was often linked to targeted therapies such as trastuzumab (anti-HER2 receptor monoclonal antibody) and was often reversible with discontinuation of the drug with recovery of cardiac function to normal [38].

### 2.8. Recovery in Anthracycline Induced Cardiotoxicity

Unlike earlier beliefs, anthracycline-induced cardiotoxicity is not always irreversible. The potential for recovery appears to be closely tied to early detection and prompt intervention:

1. Time-Dependent Recovery: An inverse relationship between the time of heart failure therapy initiation versus improvement of left ventricular ejection fraction (LVEF) was seen in a study of 201 patients with anthracycline-induced cardiotoxicity. Recovery of the LVEF at 2 months following completion of chemotherapy was 64%, while for those beyond 6 months, recovery was significantly reduced [24,38,39].
2. Early Intervention: There have been promising results (close monitoring of LVEF post-chemotherapy combined with early treatment with ACE inhibitors and beta-blockers) in LVEF after chemotherapy. When treated early, 82% of patients with cardiotoxicity achieve normalization of cardiac function in large study (n = 2625). However, only 11% of these patients regained their baseline LVEF [24,38,39], but it is likely that some degree of permanent damage remains.

### 2.9. Long Term Consequences of Permanent Damage

Despite the potential for recovery, some degree of permanent cardiac damage may persist, as follows:

1. Subclinical Damage: Despite normalization of LVEF, subclinical cardiac abnormalities may persist and continue to be at risk of future cardiovascular events [38].
2. Late-Onset Cardiotoxicity: This could mean patients may develop cardiac dysfunction years or decades even after treatment completion, that initial damage can progress overtime [24,38,39].

### 2.10. Other Oncology Drugs: Reversibility

The concept of reversibility extends beyond anthracyclines:

1. Tyrosine Kinase Inhibitors: This drug related cardiotoxicity may be partially reversible with drug discontinuation or dose reduction [23].
2. Proteasome Inhibitors: The cardiotoxicity of carfilzomib appears to be largely reversible with treatment cessation [23].

### 2.11. Implications for Management

Understanding the potential for reversibility has important implications for patient management:

1. Early Detection: Monitoring of the heart at regular intervals either during or following cancer treatment identifies changes before they become symptomatic heart failure [24,38,39].
2. Prompt Intervention: Because of this, the early initiation of cardioprotective therapies, e.g., ACE inhibitors or beta blockers, reduces mortality and improves the likelihood of cardiac function recovery [24,38,39].
3. Long-Term Follow-Up: Due to the possibility of late onset cardiotoxicity, cancer survivors require long term cardiac surveillance [24,38,39].

### 2.12. Molecular Mechanisms

The cardiotoxicity of oncology drugs is an enormously complex process with oxidative stress, inflammation, and cytokine involvement having been shown to be important in inducing cardiac dysfunction. Awareness of these processes is critical to devising strategies to avert and control cardiac problems in cancer patients [40].

Cardiotoxicity is a diverse and often drug specific phenomenon with known molecular mechanisms. For anthracyclines, one of the most well-studied cardiotoxic agents, several key mechanisms have been identified, as follows:

1. Topoisomerase 2β (Top2β) inhibition: Anthracyclines in cardiomyocytes bind to Top2β and produce DNA double strand breaks, mitochondrial dysfunction, and cell death [23,24].
2. Mitochondrial dysfunction: anthracyclines induce an effect on mitochondrial function which disrupts energy production and increases oxidative stress [23,24].
3. Calcium dysregulation: Oncology drugs may interfere with calcium homeostasis, with deleterious effects on cardiac contractility or through provoking arrhythmias [24].
4. DNA damage: The apoptosis or senescence of cardiomyocytes can be direct or indirect due to DNA damage [23].

### 2.13. Role of Oxidative Stress and Inflammation

Oxidative stress plays a central role in the cardiotoxicity of many oncology drugs, particularly anthracyclines:

1. ROS generation: Anthracyclines can redox the cycle, and hence generate ROS that damaged cellular components [23,24].
2. Antioxidant depletion: Depletions of cellular antioxidant defenses by cancer treatments can exacerbate oxidative damage [22,23].
3. Lipid peroxidation: Damage of cellular membranes, including those of mitochondria [24], can be caused by ROS induced lipid peroxidation.
4. Iron-mediated damage: One group of drugs does not tolerate being around iron, as anthracyclines can bind to iron and generate highly reactive hydroxyl radicals in the presence of oxygen [24].

### 2.14. Inflammation and Cytokine Involvement in Cardiac Dysfunction

Inflammation and cytokine signaling contribute significantly to cardiac dysfunction in cancer therapy:

1. Pro-inflammatory cytokine release: Pro-inflammatory cytokines such as TNF-α, IL-1β, and IL-6 can be released by cancer treatments directly impairing cardiac function [22,23].
2. NF-κB activation: Oncology drugs that activate NF-κB signaling induce inflammation, and may cause cardiac remodeling [23].
3. Immune cell infiltration: Myocarditis was recently reported as a consequence of some immune checkpoint therapy, including immune cell infiltration into the heart (myocardium) [22].
4. Vascular inflammation: VEGF inhibitors, for example, cause endothelial dysfunction and vascular inflammation (indirectly affecting cardiac function) [22,23].
5. Cytokine-induced contractile dysfunction: Direct cardiomyocyte contractility can be directly impaired by pro-inflammatory cytokines through various mechanisms, including alterations in calcium handling [22].
6. Fibrosis promotion: Both chronic inflammation and some cytokines promote cardiac fibrosis with long-term cardiac dysfunction [22,23].

The interplay between these mechanisms produces a rich landscape of cardiotoxicity. Inflammation is capable of causing severe oxidative stress, aggravating the situation. After cessation of the cancer therapy, this vicious cycle can result in progressive cardiac damage.

Understanding these molecular mechanisms has important implications for prevention and treatment strategies. This has included failed attempts aimed at cardiovascular protection with antioxidants or anti-inflammatory agents. Top2β’s role as a causative agent in anthracycline-induced cardiotoxicity has led to the development of Top2α selective drugs with retained anticancer activity, but reduced cardiac side effects [41].

### 2.15. Risk Factors for Cardiotoxicity

In general, patient-specific and drug-specific risk factors for cardiotoxicity can be grouped. This is important for knowing which patients are at high risk and how to monitor them and prevent problems.

#### 2.15.1. Patient-Specific Factors

Cardiotoxicity is a major risk factor for age. Patients with cancer are most susceptible to the cardiotoxic effects of cancer therapies, and these patients tend to be older. In a study of 5445 patients receiving anthracyclines, the age > 65 years was shown to be independently associated with cardiotoxicity [24]. Cardiotoxicity is much more likely if you already have pre-existing cardiovascular conditions. These patients are at high risk of developing ischemic heart disease and infarction, specifically those with hypertension, coronary artery disease, or preexisting left ventricular dysfunction. A meta-analysis of 6647 patients showed that patients with pre-existent cardiovascular disease were 2.4 times more at risk of cardiotoxicity [23].

Individual susceptibility to cardiotoxicity is highly determined by genetic predispositions. Various genetic polymorphisms that might enhance the risk of anthracycline cardiotoxicity have been characterized. For instance, increased risk [22] has been found to be associated with variants in the NADPH oxidase and doxorubicin efflux transporter genes. Specific single nucleotide polymorphisms (SNPs) in the CELF4 gene were implicated in a 2977 patient study as a predictor of anthracycline-induced cardiotoxicity [22].

Other patient-specific factors include female sex, obesity, and previous or concurrent radiation therapy to the chest. A review of 18 studies of 49,017 patients found a 1.5 fold increased risk of cardiotoxicity with the female sex [39]. Radiation-induced cardiovascular disease is an increasing concern in long-term survivors of breast cancer, lymphoma, and other malignancies who received chest-directed radiation [42]. The fibrosis caused by radiation therapy holds the potential to cause coronary artery disease, valvular disease, cardiomyopathies, arrhythmias as well as pericardial diseases, but rarely, these can be related to radiation therapy as the root cause [43].

#### 2.15.2. Drug-Specific Factors

Cardiotoxic risk is, in large part, a function of drugs. Cardiotoxicity associated with anthracyclines is the highest risk. The incidence of clinical heart failure for cumulative anthracycline dose varied from 5% to 48% in a large meta-analysis [44].

Anthracyclines are dose-limiting. With higher cumulative doses, cardiotoxicity risk is greatly increased. In accrual to doxorubicin, if cumulative doses above 400 mg/m^2^ are exceeded, the risk increases from 3 to 5% to 18 to 48% [45]. Based upon this dose dependence, recommended maximum cumulative doses of anthracyclines have been established.

Cardiotoxicity risk is also influenced by treatment duration and schedule. The longer you are exposed to cardiotoxic agents, the more likely your heart is to be damaged. Furthermore, the speed of administration of the drug can play a role in risk. For instance, slower rates of infusion of anthracyclines decrease the risk of cardiotoxicity when compared to bolus administration [21].

The risk of cardiotoxicity is greatly increased by combination therapies. For example, anthracyclines in combination with trastuzumab (anti-HER2 receptor monoclonal antibody) have shown higher rates of cardiac dysfunction than the agent alone. Anthracyclines with trastuzumab were associated with 27% increased risk of cardiac events compared with anthracyclines alone and 3–7% increased risk with trastuzumab alone.

The time when drugs are given in relation to other therapies can also affect risk. For instance, the co-administration of anthracyclines and radiation therapy to the chest has been found to be more hazardous to the heart than sequentially.

### 2.16. Clinical Manifestations

#### Symptoms and Diagnosis

Oncology drugs can cause cardiotoxicity, which can present as asymptomatic cardiac dysfunction or even severe, life-threatening cardiotoxicity. Cardiotoxicity manifests clinically with a variety of manifestations, some of which affect any component of the cardiovascular system.

Common symptoms of cardiotoxicity include the following:

1. Arrhythmias: Cardiac rhythm disturbances can be benign or potentially even fatal. They may include atrial fibrillation, ventricular tachycardia, and QT interval prolongation [23]. There may be palpitations, dizziness, or syncope.
2. Heart Failure: This is one of the most serious manifestations of cardiotoxicity. Exertional dyspnea, fatigue, peripheral edema, and reduced exercise tolerance [24] are symptoms of the disease. In severe cases patients may go as far as to develop acute pulmonary edema or cardiogenic shock.
3. Decreased Cardiac Function: This can be the first sign of cardiotoxicity, with an asymptomatic descent in left ventricular ejection fraction (LVEF). Left untreated, this can progress to symptomatic heart failure [22].
4. Myocardial Ischemia: Coronary vasospasm can occur, causing chest pain with chronic coronary syndrome or acute coronary syndrome in some oncology drugs, particularly fluoropyrimidines [23].
5. Hypertension: It is known that certain targeted therapies, notably vascular endothelial growth factor (VEGF) inhibitors, have caused or exacerbated hypertension [21].
6. Thromboembolism: Thromboembolic events such as deep vein thrombosis or pulmonary embolism [23] are increased by some chemotherapies.

Diagnosis of cardiotoxicity relies on a combination of clinical assessment, imaging studies, and biomarker analysis such as the following:

1. Echocardiography: the cornerstone of cardiac assessment of cancer patients. It enables the measurement of the LVEF, the most common parameter used to characterize cardiotoxicity. Cardiotoxicity is diagnosed when a LVEF (typically defined as <50% absolute decrease, usually >10 percentage points) is observed to decrease from >50% to <50% [22]. In particular, more advanced echocardiographic techniques such as global longitudinal strain (GLS) may detect subclinical cardiac dysfunction prior to a drop in LVEF [24].
2. Biomarkers: Troponins (troponin I and T) are highly sensitive, specific markers of myocardial injury. When episodes of elevated troponin were associated with an increased risk of subsequent cardiac dysfunction, such episodes had been reported during cancer therapy [46]. Both cardiac stress markers (BNP and NT-pro BNP) have also been shown to have utility in predicting and diagnosing cardiotoxicity [24].
3. Other Imaging Modalities: Cardiac MRI is useful for the detailed assessment of cardiac structure and function, and particularly to assess for myocarditis, a potential complication of immune checkpoint inhibitors [21]. Advanced techniques such as cardiac MRI have emerged as valuable in detecting subclinical cardiotoxicity while emerging biomarkers like specific microRNAs are under investigation for earlier detection. Cardiac function may also be assessed, and early cardiotoxicity is recorded with use of nuclear imaging techniques [47].
4. Electrocardiogram (ECG): This is not specific to cardiotoxicity but can reveal arrhythmias and conduction abnormalities that may be due to cardiac injury.
5. Endomyocardial Biopsy: It is rarely used, although it can offer definitive proof of cardiotoxicity treatment, particularly where the diagnosis cannot be otherwise determined [21].

The clinical manifestations and diagnosis of cardiotoxicity are often variable and potentially have a late onset. Cardiotoxicity can develop while some patients are treated, and others are asymptomatic even years after therapy is complete. This underscores the need for long-term cardiac surveillance in cancer survivors [22].

The important point is that cardiotoxicity needs to be detected early in order to have a chance of stopping progression towards irreversible cardiac damage. An optimal balance between the early diagnosis and monitoring of cardiotoxicity in cancer patients is optimally identified by the use of a multimodal approach that includes regular clinical assessment, imaging studies, and biomarker analysis [24,46].

### 2.17. Long-Term Consequences

Certain oncology drugs, unfortunately, can be cardiotoxic with significant long-term consequences for cancer survivors, subsequently having to transition to a risk of cardiovascular disease well into the post-treatment phase.

#### 2.17.1. Impact on Survivors’ Quality of Life

Long term challenges for survivors of cancer with cardiotoxicity are common and often impact daily life. In a cross-sectional case–control study involving 42 breast cancer survivors, those who had experienced cardiotoxicity during treatment had significantly lower left ventricular ejection fraction, global longitudinal strain, and peak oxygen consumption compared with those with and without cardiotoxicity and healthy controls [48]. The cardiotoxicity group had a mean peak oxygen consumption that was 15% lower vs. the non-cardiotoxicity group and 25% lower vs. healthy controls [48]. Reduction in survivors’ ability to perform daily activities and exercise and increase in a decreased quality of life is a result of this reduction in cardiopulmonary function.

Cardiotoxicity has long-term lasting effects that persist for years after completion of treatment. This evidence was taken from the aforementioned study, the median time of therapy was approximately 7 years, indicating the cardiopulmonary impairments observed are not transient, but rather can be long lasting [48]. This prolonged impact on physical function may contribute to increased fatigue, reduced exercise capacity, and work and social activities limitations, all of which negatively affect a survivor’s overall quality of life.

#### 2.17.2. Increased Risk of Cardiovascular Diseases Post-Treatment

Cancer survivors who have experienced cardiotoxicity as a complication of cancer treatment are at an increased risk of developing cardiovascular diseases in the years after said cancer treatment. Cellular and interstitial changes occur in the heart, caused by cardiotoxic agents such as anthracyclines [44]. These changes fall under chronic cardiotoxicity, which appears years after the end of treatment [44].

Whether the risk of late-onset cardiotoxicity is high is of particular concern. Acute cardiotoxicity, though often transitory and generally dose-independent, is contrasted by chronic cardiotoxicity, which can result in severe cardiomyopathy and even death [44]. Ventricular systolic or diastolic dysfunction is the most characteristic manifestation of chronic cardiotoxicity that can evolve to heart failure [44].

However, there were no differences in cardiovascular outcomes after six years, although those treated with potentially cardiotoxic therapies had a significantly higher risk of cardiovascular events compared to the general population. Even years after ceasing cancer treatment, this increased risk persisted, proving the need for long term cardiovascular monitoring in cancer survivors [22].

This increased cardiovascular risk has complex underlying mechanisms. For instance, anthracyclines have been shown to induce cardiomyocyte death cell non-specifically by means of apoptosis, autophagy, necrosis, necroptosis, and ferroptosis [22]. Thus, this progressive loss of cardiomyocytes combines with the heart’s low regenerative capacity, and can result in ventricular remodeling and increased propensity to cardiovascular diseases [44].

### 2.18. Management Strategies

Oncology drug-induced cardiotoxicity is a major problem in cancer treatment, therefore, a complete management strategy for preventing and treating cardiotoxicity is needed. Cardiotoxicity management involves a multimodal approach, which involves the principles of cardio-oncology into cancer care.

Recent advances in cardio-oncology emphasize structured exercise programs and nutraceutical interventions as additional strategies to mitigate cardiotoxicity. Moreover, multidisciplinary care models—where oncologists, cardiologists, and rehabilitation specialists coordinate treatment—have shown promising results [49].

For symptomatic patients, standard heart failure treatments (ACE inhibitors, beta-blockers) are used, often in combination with the temporary or permanent discontinuation of the offending agent.

#### 2.18.1. Preventive Measures

The use of cardioprotective agents is one of the key preventive strategies. The most tested cardioprotective agent is dexrazoxane in the setting of anthracycline-related cardiotoxicity. It also works as a free radical chelator, limiting the formation of free radicals that damage cardiac tissue. In patients with metastatic breast cancer who are expected to have further anthracycline treatment, dexrazoxane is recommended by the American Society of Clinical Oncology for those with cumulative doxorubicin dose greater than 300 mg/m^2^ [45]. Nevertheless, its use is constrained by apprehensions about its potential interference with antitumor efficacy, although these fears have been receded recently [38].

Early detection of cardiotoxicity depends on monitoring protocols in at risk patients. These protocols typically include the following:

1. Echocardiography and biomarker assessment (troponin, BNP) [45] baseline cardiac evaluation before starting potentially cardiotoxic therapy.
2. Left ventricular ejection fraction (LVEF) should be regularly monitored during treatment [50] with the dose depending on the specific drug and patient risks.
3. Subclinical cardiac dysfunction that can be detected before a decline in LVEF [50] and use of more delicate imaging approaches, such as quantitative global longitudinal strain (GLS) echocardiography.
4. Some forms of cardiotoxicity may not manifest for up to 15 years after completion of treatment [45] (Table 3).

#### 2.18.2. Treatment Approaches: Management of Symptomatic Patients

Management of symptomatic patients with cardiotoxicity often follows standard heart failure treatment guidelines, with some modifications:

1. For patients with reduced LVEF, the first line of treatment is with ACE inhibitors or angiotensin receptor blockers, and some evidence suggests that the drugs may also have a preventive benefit if used early in cancer treatment [38].
2. The antioxidant properties of beta-blockers, particularly carvedilol, have been shown to be useful both as a preventative as well as a treatment for chemotherapy-induced cardiotoxicity [38].
3. Referrals to cardiology services are made for patients with severe heart failure where temporary or permanent discontinuation of cardiotoxic agents may be necessary in collaboration with an oncology and cardiology team [50].

#### 2.18.3. Integration of Cardio-Oncology Principles in Cancer Care

Participation in cardio-oncology is essential for the effective management of cardiotoxicity within cancer care. This includes the following:

1. The importance of early screening for cardiovascular risk factors before initiating cancer therapy, taking into account cardiac risk factors, including potential cardiotoxicity of planned therapies [45].
2. Minimizing cardiovascular risk in cancer treatment regimes without compromising the oncological efficacy. This may use less cardiotoxic alternatives or modified dosing schedules [50].
3. Cardio-protective strategies, including exercise programs and control of cardiotoxicity risk factors [38].
4. The use of cardioprotective strategies, including ACE inhibitors, beta-blockers, and exercise programs.
5. Facilitation of communication between oncologists and cardiologists in the creation of multidisciplinary cardio oncology teams to coordinate care for a patient during the cancer treatment journey [45].
6. Survivorship care plans that include long-term cardiovascular monitoring and management strategies for cancer survivors [50], allows survivors to receive the right cancer treatment especially, long-term cardiovascular monitoring and management strategies. Long-term follow-up and survivorship care plans for cancer survivors at high risk of late-onset cardiotoxicity require the constant monitoring of patients.

### 2.19. Future Directions

Cardio-oncology is an extremely new field of study that is progressing rapidly, and there is ongoing study to fill the significant gaps in our knowledge of oncology drug induced cardiotoxicity. Ongoing research is exploring the mechanisms, genetic risk factors, and novel therapies to better predict and prevent cardiotoxicity. The integration of artificial intelligence and machine learning for risk prediction is a promising avenue that could refine early detection and personalized treatment strategies. Additionally, regenerative medicine approaches—such as stem cell therapies—are being investigated to repair long-term cardiac damage. Further studies on adjunct therapies, including advanced nutraceuticals and exercise interventions, are also warranted.

#### 2.19.1. Gaps in Understanding Mechanisms and Risk Factors

For many of these oncology drugs, those important gaps in our understanding of the precise mechanisms of cardiotoxicity remain despite significant advances. For example, the activity of topoisomerase 2β (Top2β) in anthracycline induced cardiotoxicity is well documented, but not the mechanisms of cardiotoxicity of newer targeted therapies [23].

Much research is currently devoted to the identification of genetic and molecular biomarkers that are useful for predicting individual susceptibility to cardiotoxicity. Although several genetic polymorphisms for increased risk of anthracycline induced cardiotoxicity have been identified [22], the clinical utility of these polymorphisms has yet to be established. Future research should be directed at ensuring that high risk patients are identified more accurately through the construction of complete predictive risk models based on genetic, molecular and clinical risk factors [51].

#### 2.19.2. Importance of Personalized Medicine in Oncology

Personalized medicine appears to be coming of age in cardio-oncology. Flipping this around, cancer treatments could be tailored to factors that influence both efficacy and cardiotoxicity in each individual patient: genetic profile, preexisting cardiovascular risk factors, and tumor biology, reducing cardiotoxicity plus maximizing efficacy [23]. To develop personalized risk assessment and treatment strategies, this approach needs to integrate multi-omics data (genomics, proteomics, metabolomics) into clinical information.

The development of precision cardio-oncology approaches may involve the following:

1. Testing of patients at higher risk for cardiotoxicity, using pharmacogenomic testing.
2. Individual risk-based monitoring protocols.
3. Targeted cardioprotective agents use, such as dexrazoxane, and personalized cardioprotective strategies.
4. Balanced oncological efficacy and cardiovascular risk for individualized cancer treatment regimens.

#### 2.19.3. Innovative Therapies

The key research involves inventing new drugs with reduced cardiotoxic profiles. A highly promising strategy is to design anthracycline analogs selective for Top2α (the sole target in cancer cells) and inducing the minimum level of Top2β (the promoter of cardiotoxicity) [23]. Targeted approaches of this nature, however, could maintain anti-tumor efficacy while minimizing cardiac side effects.

#### 2.19.4. Development of New Drugs with Reduced Cardiotoxic Profiles

The development of another innovative direction has been the exploration of drug delivery systems based on nanotechnology. We have identified anthracycline liposomal formulations as a promising example of delivery vector modulations that can preserve anti-tumor efficacy but reduce cardiotoxicity [2]. Future research into nanoparticle-based delivery systems may enhance targeting of anticancer drug delivery, minimizing cardiac tissue exposure to toxic agents [52].

#### 2.19.5. Exploration of Adjunct Therapies to Mitigate Cardiac Risks

Active research also examines adjunct therapies that will help reduce cardiac risks.

These include the following:

1. Cardioprotective agents: Researchers are also studying other potential cardioprotective agents, such as antioxidants, iron chelators and modulators of cellular signaling pathways that have been implicated as sources of toxicity [53].
2. Exercise interventions: Evidence is emerging for cardio protection associated with structured exercise programs during cancer treatment. More research is needed to find out how to best optimize exercise and who is best likely to benefit [23].
3. Nutraceuticals and dietary interventions: Other studies have attempted to minimize the cardiotoxicity potential of nutraceuticals (such as coenzyme Q10 and L-carnitine). Although preliminary results are promising, larger clinical trials are required to establish efficacy and safety [54].
4. Cell-based therapies: Cardiac tissue damaged by cancer treatments is being investigated to repair and regenerate through stem cell therapies and exosome-based approaches [55].

## 3. Discussion

Analyzing the above-mentioned clinical studies, it can be stated that cardiotoxicity rates vary greatly across different agents. Nearly 25% of all cancer patients may experience some form of cardiotoxicity, and the risk of cardiac mortality for pediatric cancer survivors is eightfold greater than in the general population. The need for cardiovascular monitoring both during and after cancer treatment is underscored.

The management of cardiotoxicity is dependent on the close collaboration of oncologists and cardiologists, and patients are frequently not referred to cardiology until symptoms have arisen. The reason that only 15% were referred prior to chemotherapy suggests a considerable decrease in preventive care. Cancer therapies have, however, progressed: survival rates have improved-up from 50% in the 1970s to 67% in recent years, but these treatments come at a price-all, leading to long-term quality of life issues, such as fatigue, pain, and cognitive problems.

Interestingly, early detection and intervention for anthracycline-induced cardiotoxicity is not always irreversible, and cardiac function can be recovered. Timely treatment improves heart function in many suspected patients. But factors such as age, pre-existing cardiovascular conditions, and genetic grounds also play a role in who may or may not be susceptible to cardiotoxicity. To protect patients’ cardiovascular health from cancer treatment, studies stress the need for ongoing research into these risks and into developing effective preventative strategies and point towards a new science, “cardio-oncology”, where the central role is represented by personalized medicine studies.

## 4. Conclusions

The cardiac toxicity of oncology drugs is a significant issue in the management of cancer. In addition, the economic burden of cardiotoxicity and the importance of patient education and shared decision-making should not be overlooked. Potential solutions could be the integration of cardio-oncology practices, in which oncologists and cardiologists collaborate to assess heart related risks prior to and during cancer treatment. This joint work may also result in the creation of new guidelines factoring cardiovascular health into cancer treatment decisions. The novel biomarkers might enable the identification of cardiac injury upon presentation, allowing for timely intervention. Understanding and using cardioprotective agents is essential in order to reduce the risk of cardiac injury without diminishing the efficacy of oncologic therapies. Stem cell therapies can be beneficial in safeguarding patients from long-term side effects of chemotherapy.

Therefore, it can be concluded that the treatment of oncology patients necessitates a holistic view of the risks inherent to cancer management, where the key to success will be the fusion of cardiology and oncology specialties.

## Figures and Tables

**Table 1:** Main classes of chemotherapy medication and their proven mechanisms of cardiotoxicity.

Class of Chemotherapy Medication	Mechanism of Cardiotoxicity
Anthracyclines	Anthracyclines cause cardiotoxicity primarily via oxidative stress, mitochondrial dysfunction, and DNA damage.
Tyrosine Kinase Inhibitors (TKIs)	TKIs disrupt normal cellular signaling and can impair mitochondrial function, leading to cardiomyocyte apoptosis.
Immune Checkpoint Inhibitors	Recent findings have revealed that immune checkpoint inhibitors not only enhance anti-tumor immunity but also may induce myocarditis, arrhythmias, and other immune-related cardiac events.
Anti-Angiogenic Drugs (anti-VEGF)	Inducing microvascular rarefaction, reducing myocardial capillary density, and impairing cardiac perfusion.
Anti-Angiogenic Drugs (anti-PIGF)	Safer than anti-VEGF.

**Table 2:** Summary of Oncology Drug-Induced Cardiotoxicity Types.

Type of Cardiotoxicity	Description	Common Oncology Drugs Involved	Potential Long-Term Effects
Left Ventricular Dysfunction and Heart Failure	Progressive decline in heart’s ability to pump blood effectively.	Anthracyclines (e.g., doxorubicin), Anti-HER2 agents (trastuzumab).	Chronic heart failure, reduced ejection fraction.
Arrhythmias	Irregular heartbeats, which may be benign or life-threatening.	Tyrosine kinase inhibitors (TKIs), Immune checkpoint inhibitors.	Increased risk of sudden cardiac events, atrial fibrillation.
Myocardial Ischemia	Reduced blood flow to the heart muscle, leading to chest pain and infarction.	Fluoropyrimidines (e.g., 5-FU), VEGF inhibitors.	Coronary artery disease, increased risk of heart attacks.
Hypertension	Elevated blood pressure due to vascular dysfunction.	VEGF inhibitors, certain TKIs.	Increased risk of stroke and heart failure.
Thromboembolism	Formation of blood clots that can lead to deep vein thrombosis or pulmonary embolism.	VEGF inhibitors, Immunotherapy agents.	Pulmonary embolism, increased cardiovascular mortality.
Pericardial Disease	Inflammation or fluid accumulation around the heart.	Radiation therapy, certain targeted therapies.	Pericardial effusion, chronic pericarditis.

**Table 3:** Preventive Strategies and Treatment Approaches for Cardiotoxicity.

Strategy	Description	Application
Baseline Risk Assessment	Cardiovascular screening before initiating cancer therapy.	Identifying high-risk patients based on comorbidities, prior cardiac history.
Regular Cardiac Monitoring	Routine imaging and biomarker assessment.	Echocardiography (LVEF, GLS), troponin levels, BNP/NT-proBNP.
Cardioprotective Medications	Use of ACE inhibitors, beta-blockers, and dexrazoxane.	Preventing or mitigating drug-induced cardiotoxicity.
Modified Drug Administration	Adjusting dosage, infusion rates, or drug combinations.	Reducing peak plasma concentrations of cardiotoxic drugs.
Exercise and Lifestyle Interventions	Structured exercise programs and dietary interventions.	Improving cardiac resilience and mitigating treatment effects.
Early Intervention and Treatment	Initiating heart failure treatments at the first sign of dysfunction.	Improving recovery rates and reducing progression to irreversible damage.
Multidisciplinary Cardio-Oncology Collaboration	Close coordination between oncologists and cardiologists.	Optimizing both cancer therapy and cardiac protection.
